# The effects of vitamin D administration on brain inflammatory markers in high fat diet induced obese rats

**DOI:** 10.1186/s12868-017-0400-1

**Published:** 2017-12-28

**Authors:** Mahdieh Abbasalizad Farhangi, Mehran Mesgari-Abbasi, Ghazaleh Nameni, Ghazaleh Hajiluian, Parviz Shahabi

**Affiliations:** 1 Drug Applied Research Center, Nutrition Research Center, Department of Community Nutrition, Tabriz University of Medical Sciences, Attar Neyshabouri Street, Tabriz, Iran; 2 Student Research Committee, Tabriz University of Medical Sciences, Tabriz, Iran; 3 Nutrition Research Center, Department of Community Nutrition, Tabriz University of Medical Sciences, Tabriz, Iran; 4 Drug Applied Research Center, Tabriz University of Medical Sciences, Tabriz, Iran

**Keywords:** NF-Kβ, IL-6, IL-1β, Acetylcholine, Vitamin D, Obesity, HFD

## Abstract

**Background:**

Obesity induced brain inflammation is associated with cognitive disorders. We aimed to investigate the influence of vitamin D on hypothalamus and hippocampus inflammatory response in high-fat diet induced obese rats.

**Methods:**

In the beginning of the study, 40 rats were divided into two groups: control diet and high fat diet (HFD) for 16 weeks; then each group subdivided into two groups including: N, ND + vitamin D, HFD and HFD + vitamin D. Vitamin D supplementation was done for 5 weeks at 500 IU/kg dosage. IL-6, IL-1β, NF-Kβ and acetylcholine (ACH) and brain derived neurotropic factor (BDNF) concentrations in hippocampus and hypothalamus homogenate samples were measured by commercial ELISA kits.

**Results:**

Vitamin D administration, reduced food intake and weight gain in studied groups (*P* < 0.001). Vitamin D reduced hippocampus acetylcholine concentrations in ND + vitamin D group (*P* < 0.001). High fat diet increased hippocampus IL-6 concentrations significantly (*P* < 0.05) compared with normal diet receiving groups. Vitamin D could not have significant effects on IL-6 concentrations. Vitamin D administrations reduced IL-1β, NF-Kβ and acetylcholine concentration and BDNF concentrations in ND + vitamin D compared with ND group. These reductions were not significant in HFD + vitamin D versus HFD group.

**Conclusion:**

According to our results, vitamin D reduced food intake and weight gain and modulated the HFD induced inflammatory response in hippocampus and hypothalamus of high fat diet induced obesity. Therefore, this neurosteroid, can be suggested as a supplemental therapeutic tool in prevention of obesity related cognitive and neurodegenerative problems.

## Background

Obesity is a major health problem worldwide and is associated with numerous chronic disease including cardiovascular disease, diabetes, metabolic syndrome and even some types of cancers [1]. The prevalence of obesity is growing rapidly and according to the WHO estimation, in 2014, more than 1.9 billion adults, 18 years and older, were overweight. Of these over 600 million were obese [2]. High-fat diet has been shown to influence eating behavior and is make susceptibility in both human and animal models in developing obesity [3]. Moreover, because of the great similarity between the genomes of rodents and humans, this animal model is a very useful tool for studying obesity [4].

Obesity has been regarded as a chronic low-grade inflammatory condition and this increased peripheral inflammatory tone, will also increase the occurrence of central nervous system (CNS) inflammation [5]. The increased inflammation in CNS, involves greatly in the pathogenesis of CNS-related disease including dementia, Alzheimer disease (AD) and stroke [6]. More important regions of CNS extensively studied to be susceptible in HFD-induced damages are hypothalamus and hippocampus [7–9]. Hypothalamus is especially attracted much attention because of its pivotal role in food intake regulation and availability of nutrients [10]. Hypothalamus inflammatory response after dietary fat-induced obesity is an important contributor in developing insulin and leptin resistance and defective food intake [11, 12]. High fat diet feeding is associated with a disruption of the homeostasis in the hypothalamus and increased inflammatory response due to glial cell activation [13]. The inflammatory response to dietary fat and especially dietary saturated fatty acids in the hypothalamus is mediated by toll-like receptors (TLRs) which their activation and signaling by dietary fat leads to activation of nuclear factor kappa-β (NF-Kβ) and production of inflammatory cytokines including interleukin (IL)-1β and IL-6 [14]. Accordingly, another important brain region, hippocampus, is also susceptible to inflammation in obesity and numerous studies have revealed that neural systems of hippocampus involved in memory and cognition are also negatively affected in obesity [15]. Although numerous mechanisms such as change in gut peptides and reduced neurotrophic factors are suggested, increased pro-inflammatory cytokines like IL-6 might also be important contributor in hippocampal injury in obesity [16]. Moreover, clinical evidence suggests that inflammation in the brain and hippocampus of HFD-fed mice is regulated by IL-6 that has a main function in cognitive performance like learning and memory [17].

Recently it has been proposed that there is a link between acetylcholine and obesity or insulin resistance [18, 19] and the potential of muscarinic acetylcholine receptors as a therapeutic target of obesity has been proposed [20, 21]. This is probably because of altered acetylcholine turn-over in high fat diet induced obesity; previous studies suggest that choline deficient diets prevents high fat induced obesity in rats [18]. The suggested mechanism is the conversion of choline to acetylcholine in neurons. Under normal conditions the availability of choline for acetylcholine production in cholinergic neurons is controlled by a high affinity choline transporter, which is normally saturated with choline [22, 23]. However, when acetylcholine turnover is high, choline supply appears to become limiting for the production of acetylcholine [24]. Furthermore, brain choline and acetylcholine levels are lower in rats fed diet choline-deficient diets than in rats fed a choline-rich diet [25]. The M_3_ muscarinic acetylcholine subtype receptor is important in regulating energy metabolism [26]. Mice that lacked the M_3_ receptor displayed increased energy expenditure, were protected from obesity, and showed increased insulin sensitivity. Because the M_3_ receptor is expressed primarily in the central nervous system, and not in tissues (such as muscle, adipose and liver) that play major roles in glucose and lipid metabolism, it was suggested that activation of the M_3_ receptor in the central nervous system by acetylcholine is required for obesity to develop on a HF diet [26]. Taken together, these data indicate that altered acetyl choline metabolism plays a central role in the high fat diet induced obesity and its related disorders.

Considering the above-mentioned introduction, the role of obesity and high-fat diet in development of brain inflammation and triggering the obesity-related neural disorders are well elucidated; however, much less is known about the responsiveness of this pathogenic phenomenon to therapeutic agents especially brain-influential nutrients. Vitamin D classically is known as a steroid hormone responsible in regulating calcium homeostasis, in addition, it can also involve in brain functions because of its capability in passing through blood brain barrier (BBB) and acting on CNS via its receptors. Vitamin D can preserve cognition including attention, memory orientation, executive function [27, 28] and inhibits brain dysfunction in disease models of multiple sclerosis [29] and AD [30]. It also acts as an anti-inflammatory, antioxidant and neuro-protective steroid hormone [31]. However, no study is available evaluating the role of this vitamin in obesity-induced brain inflammation; therefore, the primary hypothesis of the current experimental study was to investigate the influence of vitamin D on brain derived neurotropic factor (BDNF) and neuro-inflammatory factors including IL-6, IL-1β, NF-Kβ and acetylcholine (ACH) with especial regard to hypothalamus and hippocampus in high-fat diet induced obese rats. The secondary hypothesis was to investigate the effects of vitamin D on food intake and weight gain in these animals.

## Methods

The design of study has been mentioned in our previous report [32, 33]. Therefore, the animals and procedures are reported here briefly.

### Animals, diets and experimental protocol

Forty male Wistar rats weighted 200–220 g were purchased from the Pasteur institute animal care center (Karaj, Iran) and were housed five in each cage under standard conditions (light on from 07:00 AM to 07:00 PM and constant temperature of 25 ± 2 °C) with ad libitum access to standard laboratory chow diet and water. After a week, animals were randomly assigned into 2 groups (n = 20, each group) of normal diet (ND) or high fat diet (HFD). ND included 10% fat, 30% protein and 60% carbohydrate and HFD with 59% fat, 11% protein and 30% carbohydrate. After 4 months of receiving these diets, groups were randomized into two sub-groups according to vitamin D or vehicle administration as follows: ND, ND + vitamin D, HFD and HFD + vitamin D. Migliol (Sigma Adrich, USA) was used as vehicle and vitamin D dosage was 500 IU/kg/d administered by oral gavage. The duration of this phase of study was 5 weeks.

The rationale of vitamin D dose and duration was according to the previous studies confirming the neuro-protective role of this dosage of vitamin in animal models [34].

Body weight was weekly measured by scale (PAND Industries, px3000, 5 kg ± 1 g) and food intake was monitored 3 times a week. Daily food intake was recorded in a metabolic cage until sacrifice. Briefly, five rats from each group were housed per cage and amount of remaining food from past 24 h was weighed every day. All of the experiments were conducted in accordance with the National Institutes of Health (NIH) ethical guidelines for the care and use of laboratory animals (NIH; Publication No. 85-23, revised 1985) and was approved by the veterinary ethics committee of the Tabriz university of medical sciences (Registration number: TBZMED.REC.1395.532).

### Preparation of blood, hippocampus and hypothalamus samples

After an overnight fasting, the rats were anesthetized with Ketamin (6.6 mg/kg) and Xylazine (0.3 mg/kg) intra peritoneally. Blood samples were obtained from cardiac puncture and centrifuged at 10,000 g at 4 °C for 20 min; sera were separated and stored in an ultra-low temp freezer (Jal Tajhiz Production, Iran) at − 80 °C until assaying. After rats were sacrificed by decapitation, their brains were removed and the hippocampus was dissected. Accordingly, hypothalamus was also located and isolated according to brain control planes and the hippocampus hemispheres and hypothalamus samples were immediately stored at − 80 °C until further use. For assay, the hippocampal and hypothalamus tissues were homogenized in phosphate buffered saline (PBS) and centrifuged at 10,000 g at 4 °C for 20 min, and clear supernatants were collected for assessment of inflammatory parameters by ELISA assay.

### ELISA

Before and after vitamin D supplementation, initial and terminal serum vitamin D concentrations was measured by individual enzyme-linked immunosorbent assay kit (ELISA) (Eastbiopharm, Zhejiang, China) according to the manufacturer’s instructions. The inter and intra assay coefficient of variation for vitamin D was < 10% and sensitivity was 0.95 ng/ml. Accordingly, measurements of inflammatory cytokines including IL-6, IL-1β, NF-Kβ and acetylcholine (ACH) and brain derived neurotropic factor (BDNF) in hippocampus and hypothalamus homogenate samples were performed by commercial ELISA kit (Hangzhou Eastbiopharm, Zhejiang, China). The inter and intra assay coefficient of variation for ACH were < 12% and sensitivity was 0.52 u/ml. The corresponding values for BDNF were < 10, < 12% and 0.01 ng/ml, for IL-6, < 12% and 2.49 ng/L, for IL-1β < 12% and 10.23 pg/L and for NF-Kβ were < 10, < 12% and 0.023 ng/ml.

### Statistical analysis

All statistical analyses were performed using SPSS software, version 16. Kolmogorov–Smirnov test was performed for normality of the distributions of variables. Data are expressed as the mean ± SD. The data were analyzed using one-way analysis of variance (ANOVA) with 4 levels followed by post hoc Tukey test for comparisons between multiple groups. Paired sample *t* test was used for before and after comparison of parameters. Repeated measures test was used for comparison on intra-group changes in body weight and food intake. *P* < 0.05 was considered as statistically significant.

## Results

### Changes in food intake, body weight and serum vitamin D concentrations during the study period

**Fig. 1:**
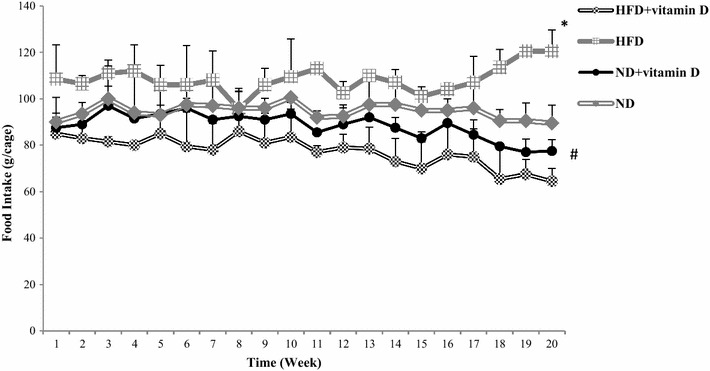
Vitamin D and food intake of rats with normal diet (ND), normal diet supplemented with vitamin D (ND + vitamin D), high fat diet (HFD) and high fat diet supplemented with vitamin D (HFD + vitamin D). Data are expressed as mean ± SD. (*P = 0.11 vs. ND; ^#^ *P* = 0.008 vs. HFD)

Changes in food intake of animals during the study period have been shown in Fig. 1. Food intake of animals was gradually increased until the administration of vitamin D and this increase was meaningful in all of study groups. However, after vitamin D administration, in ND and HFD groups, food intake decreased significantly (*P* < 0.001). Accordingly, body weight of all of studied groups increased significantly and HFD led to a significant weight gain compared with ND as published in our previous report (Table 1). However, after vitamin D administration, a gradual reduction in body weight has been observed. The results of the trend of body weight have been published elsewhere [23, 24]. As shown in Table 1, the results of repeated measure analysis showed that in each group significant changes in the weight has been occurred. By the way, the results of one way ANOVA and post hoc *Tuky* test in between group comparisons showed that the significant difference in comparison of the weight of 16th week was between ND versus HFD and HFD + Vit D, ND + Vit D versus HFD and HFD + Vit D and HFD versus ND. Whereas in comparison of weight of 21st week between groups all of the inter group comparisons were significant. Moreover, vitamin D administrations led to a marked increase in serum vitamin D concentrations in ND + vitamin D and HFD + vitamin D groups (Table 2). According to the results of one way—ANOVA and its followed post hoc *Tukey* test, the significant difference in inter-group comparisons of serum vitamin D concentrations between ND versus ND + Vit D and HFD + Vit D, ND + Vit D versus HFD and HFD versus HFD + Vit D. [23, 24].

**Table 1:** Changes in body weight of rats

Groups	1st week	16th week	21st week	*P* value^†^
ND	219 ± 10.83	276 ± 26.72	289 ± 29.80	0.001
ND + vitamin D	225 ± 22.09	278 ± 27.38	256 ± 26.90	0.001
HFD	219 ± 13.27	403 ± 4.13	425 ± 3.71	0.001
HFD + vitamin D	225 ± 13.77	340 ± 8.7	381 ± 7.80	0.001
F values	0.170	135.097	147.026	
^‡^ *P* value	0.69	0.001	0.001	

Data are expressed as mean ± SD. Statistical differences between groups were assessed by one-way ANOVA followed by *Tukey’s* test for *Post Hoc* analysis. Intra group comparisons of body weight were performed by repeated measure analysis

*HFD* high fat diet, *ND* normal diet

^†^ *P* value and ^‡^ *P* value indicated intra group and inter group differences respectively. ^‡^The significant difference in comparison of the weight of 16th week was between ND versus HFD and HFD + Vit D, ND + Vit D versus HFD and HFD + Vit D and HFD versus ND. Whereas in comparison of weight of 21st week between groups all of the inter group comparisons were significant. *P* < 0.05 was considered as statistically significant. N = 20 in each group

**Table 2:** Vitamin D concentrations in study groups

Groups	16th week	21th week	Mean difference*	*P* value^†^
ND	47.5 ± 7.32	36.3 ± 7.74	− 11.20 ± 1	0.001
ND + vitamin D	54.9 ± 11.53	119 ± 26.01	64.2 ± 7.8	0.001
HFD	51.2 ± 11.16	37.7 ± 11.53	− 13.5 ± 4.25	0.01
HFD + vitamin D	53.2 ± 14.11	124 ± 39.39	70.5 ± 12.85	0.001
F values	0.792	39.272	–	–
^‡^ *P* value	0.50	0.001	–	–

Data are expressed as mean ± SD. Statistical differences between groups were assessed by one-way ANOVA followed by Tukey’s test for *Post Hoc* analysis. Intra group comparisons of vitamin D concentration were performed by paired t-test analysis

*HFD* high fat diet, *ND* normal diet

^†^ *P* value and ^‡^ *P* value indicated intra group and inter group differences, respectively. ^‡^The significant difference is between ND versus ND + Vit D and HFD + Vit D, ND + Vit D versus HFD and HFD versus HFD + Vit D

*Mean difference was calculated by subtracting the weight of 16th from weight of 21st weeks. *P* < 0.05 was considered as statistically significant. n = 20 in each group

### Vitamin D administration and neuro-inflammatory parameters in the hippocampus

**Fig. 2:**
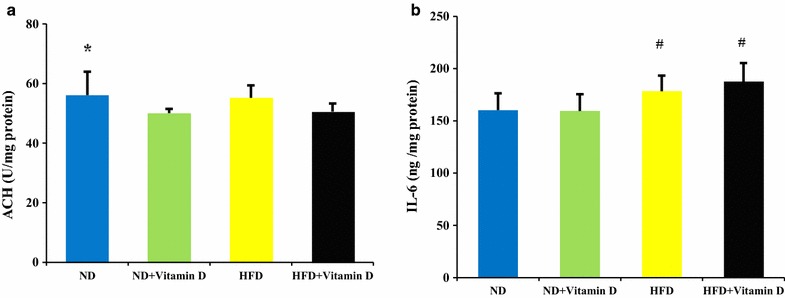
Acetylcholine (ACH) and IL-6 concentration in hippocampus of studied groups. ND, normal diet; HFD, high fat diet. Data are expressed as mean ± SD. Statistical differences between groups were assessed by one-way ANOVA followed by post hoc Tukey’s test for post hoc analysis.*P < 0.05 versus ND + Vit D. ^#^ *P* < 0.05 versus ND and ND + Vit D. F values of one way ANOVA for **a** and **b** were 3.34 and 4.099 respectively. *P* < 0.05 was considered as statistically significant. n = 20 in each group

The effects of vitamin D administration on hippocampus acetylcholine and IL-6 concentrations are presented in Fig. 2. Vitamin D reduced hippocampus acetylcholine concentrations in ND + vitamin D group compared with ND group (*P* < 0.001). Whereas, reduced acetylcholine concentrations in HFD + vitamin D group compared with HFD group was not significant. Moreover, high fat diet increased hippocampus IL-6 concentrations significantly in HFD and HFD + Vit D groups (*P* < 0.05) compared with ND and ND + Vit D groups. Vitamin D could not have significant effects on IL-6 concentrations.

### Vitamin D administration and neuro-inflammatory parameters in the hypothalamus

**Fig. 3:**
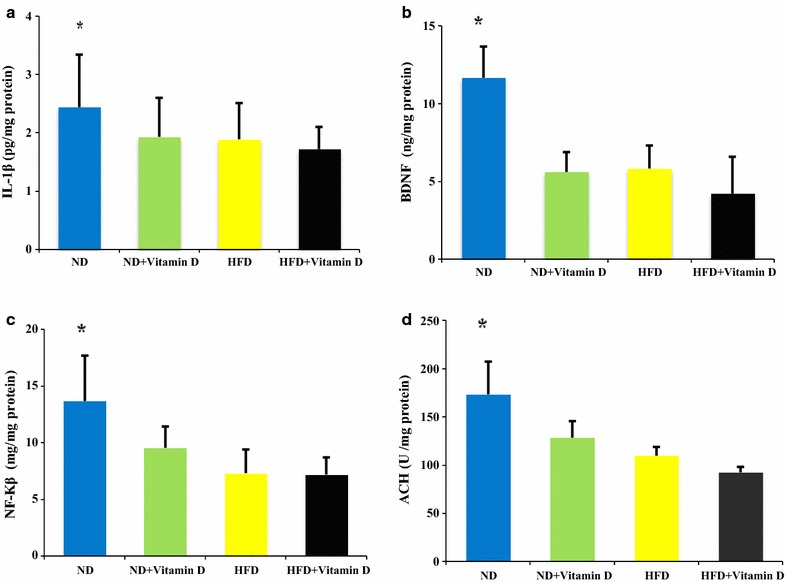
IL-1β, BDNF, NF-Kβ and acetylcholine (ACH) concentration in hypothalamus of studied groups. ND, normal diet; HFD, high fat diet. Data are expressed as mean ± SD. Statistical differences between groups were assessed by one-way ANOVA followed by post hoc *Tukey’s* test for post hoc analysis.*P < 0.05 versus ND + Vit D, HFD and HFD + Vit D. F values of one way ANOVA for **a**, **b**, **c** and **d** were 4.12, 3.57, 4.57 and 3.44 respectively. *P* < 0.05 was considered as statistically significant; n = 20 in each group

Among inflammatory parameters in hypothalamus, vitamin D administrations reduced IL-1β, NF-Kβ and acetylcholine concentration and BDNF concentrations in ND group compared with ND + vitamin D, HFD and HFD +Vit D (*P* < 0.05). These reductions were not significant in HFD + vitamin D versus HFD group (Fig. 3).

## Discussion

In the current study, vitamin D reduced food intake and weight in ND +vitamin D and HFD + vitamin D compared with ND and HFD rats; moreover, vitamin D reduced ACH concentrations in hippocampus of ND + vitamin D compared with ND group. Hippocampus IL-6 in HFD receiving groups was significantly higher than ND receiving groups while vitamin D had no significant effect on its concentrations. Also, the IL-1β, BDNF, NF-Kβ and ACH in hypothalamus of ND groups was higher than other groups. Vitamin D reduced IL-1β, BDNF, NF-Kβ and ACH concentrations in hypothalamus of ND compared with ND + vitamin D group. The reduction of these inflammatory parameters in HFD + vitamin D group were not statistically significant.

Our hypothesis about the effects of vitamin D on food intake and weight has been accepted; reduced food intake after vitamin D administration which also led to reduced weight in HFD + vitamin D and ND + vitamin D compared with HFD and ND groups was also confirmed in previous animal or human studies [35, 36]. In the study by Major GC [36] vitamin D supplementation reduced weight and fat mass and lipid intake in females. The suggested underlying mechanisms is that increased calcium absorption leads to reduced intra-adipocyte lipogenic gene expression and stimulation of lipolysis and adipocytes uncoupling protein 2 expression [37, 38]. It has been suggested that vitamin D deficiency increases appetite and decreases energy consumption by stimulating Agouti Related Protein/Neuropeptide Y (AgRP/NPY) and suppressing the pro-Opiomelanocortin/Cocaine-Amphetamine-Regulated Transcription (POMC/CART) pathway [39].

According to our results, vitamin D administrations led to a significant reduction in neuro-inflammatory factors including IL-1β, BDNF, NF-Kβ and ACH in hypothalamus and ACH in the hippocampus of ND +vitamin D group compared with ND group. Whereas, this reduction, was not significant in HFD + vitamin D. In other word, reduced neuro-inflammatory factors after vitamin D administrations in normal diet receiving rats was more pronounced compared with high fat receiving rats. The possible underlying mechanism is that obesity leads to decreased vitamin D bioavailability via several possible mechanisms including trapping vitamin D in fat mass, decreased vitamin D biosynthesis by reduced physical activity and reduced exposure to sunlight and hepatic steatosis and reduced 25-hydroxy vitamin D synthesis in obese individuals [40].

The effects of vitamin D in reducing neuro-inflammatory parameters in hypothalamus like IL-6, IL-1β and NF-Kβ further confirms the anti-inflammatory effects of this vitamin. Recent studies showed that vitamin D and its analogues—1, 25(OH)_2_D_3_ and 25(OH)D_3_—inhibits lipopolysaccharide-induced p38 phosphorylation, IL-6, and TNFα production by human monocytes in a dose-dependent manner; moreover, 1,25(OH)_2_D_3_ or its analogs reduce monocyte chemoattractant protein (MCP)-1 and IL-6 expression via inhibiting NF-κB activation in macrophages [41, 42]. Accordingly, the potential ability of vitamin D for crossing through the BBB and activation of its receptors in brain cells exerts its direct impact in the CNS [43]. The involvement of vitamin D in the function of the central nervous system is supported by the presence of the enzyme 25(OH)D_3_-1α-hydroxylase, responsible for the formation of the active form of vitamin D, as well as the presence of vitamin D receptors in the brain, mainly in the hypothalamus and dopaminergic neurons of the substantia nigra [44]. The anti-inflammatory actions of vitamin D against neuro-degenerative disease like multiple sclerosis and schizophrenia has been studied before [45, 46].

Reduced acetylcholine synthesis in hippocampus and hypothalamus of rats in the current study after vitamin D supplementation was also approved in previous reports; vitamin D has been introduced as an important factor modifying the synthesis of several neuro-mediators like acetylcholine via increased gene expression of the enzyme choline acetyl-transferase (CAT) [47, 48]. This action all confirms the neurosteroid actions of vitamin D as previously suggested by Kalueff [49]. As previously suggested, the activation of the M_3_ muscarinic acetylcholine subtype receptor in the central nervous system by acetylcholine is required for obesity to develop on a high fat diet [26]. In choline deficient diets obesity did not develop because of lower CNS acetylcholine supply [25]. Choline deficient animals are protected against obesity and vitamin D also regulated this pathway by lowering acetyl choline supply in the brain [25].

Brain derived neurotropic factor (BDNF), is a neurotropic hormone that plays a fundamental role in development and plasticity of the central nervous system (CNS). It is currently recognized as a major participant in appetite control and food intake [50]. Previous studies reported the increased BDNF concentration in obesity and its major role in disturbed glucose metabolism [50–52] and increased BDNF gene expression in adipose tissue of high-calorie diet induced obese mice [53]. In the current study, vitamin D administrations also reduced BDNF concentrations in ND + vitamin D group. Same as our results, in a study by Pozzi et al. [27] vitamin D supplementation, diminished BDNF expression and its plasma concentrations in postmenopausal women. In other study, consistently, vitamin D administrations decreased exercise-induced BDNF in rat hippocampus and abolished the BDNF downstream signal transduction cascade important for learning and memory. The authors suggested that BDNF is a vitamin D receptor (VDR) regulated protein affected by VDR suppression; this results can be explained by the fact that vitamin D supplementation in this issue plays a crucial role in the modulation of BDNF in a compensatory mechanism [27, 54–56].

In conclusion, the current study revealed the potential anti-inflammatory effects of vitamin D administration in hippocampus and hypothalamus and its modulating effects on BDNF and acetyl choline in high fat-diet induced obese rats. Therefore, this neurosteroid, can be suggested as a supplemental therapeutic tool to prevent obesity induced CNS-related neurodegenerative problems and inflammation-related cognitive disorders in obesity. Although, because of the animal model of the study, more studies in human models are warranted for further confirming the findings.
